# Bioelectrochemical treatment of municipal solid waste landfill mature leachate and dairy wastewater as co-substrates

**DOI:** 10.1007/s11356-020-10167-7

**Published:** 2020-07-22

**Authors:** Silvia Bolognesi, Daniele Cecconet, Arianna Callegari, Andrea G. Capodaglio

**Affiliations:** 1 Department of Civil Engineering and Architecture, University of Pavia, 27100 Pavia, Italy; 2 LEQUiA, Institute of the Environment, Universitat de Girona, 17003 Girona, Spain; 3 Department of Chemistry, University of Pavia, 27100 Pavia, Italy

**Keywords:** Bioelectrochemical systems, Bioelectricity, Leachate treatment, Agrofood industry wastewater, Electrode materials

## Abstract

Despite solid wastes’ landfill disposal limitation due to recent European legislation, landfill leachate disposal remains a significant problem and will be for many years in the future, since its production may persist for years after a site’s closure. Among process technologies proposed for its treatment, microbial fuel cells (MFCs) can be effective, achieving both contaminant removal and simultaneous energy recovery. Start-up and operation of two dual-chamber MFCs with different electrodes’ structure, fed with mature municipal solid waste landfill leachate, are reported in this study. Influent (a mix of dairy wastewater and mature landfill leachate at varying proportions) was fed to the anodic chambers of the units, under different conditions. The maximum COD removal efficiency achieved was 84.9% at low leachate/dairy mix, and 66.3% with 7.6% coulombic efficiency (CE) at a leachate/dairy ratio of 20%. Operational issues and effects of cells’ architecture and electrode materials on systems’ performance are analyzed and discussed.

## Introduction

Municipal solid waste (MSW) disposal is a problem with no easy or unique solution. In 2015, 242.3 Mt of MSW was produced in the European Union, 62 Mt of which discarded in landfills. Italy, in this context, produced about 29.5 Mt MSW in 2015, of which 7.8 landfilled (ISPRA 2017). Despite the reduction of MSW landfill disposal due to recent European legislation (EU 2018a; EU 2018b), leachate generated from decomposition of MSW in landfills is still a significant problem nowadays and will be for many years in the future, since its production may persist for years after a site’s closure. The risk of groundwater pollution by leachate spills from damaged landfill containment is significant, and specific monitoring is normally required in these situations due to the possible spread of harmful pollutants (Capodaglio et al. 2016a).

Leachate characteristics are quite variable, affected by landfill construction and age, local meteorology, waste type, and composition, normally high in COD and ammonia content (Kulikowska and Klimiuk 2008; Youcai 2018). Typically, a leachate’s BOD/COD ratio decreases from around 0.7 to 0.04 with landfill aging (Sonawane et al. 2017), becoming less suitable to biodegradation in time. Leachate contains organic constituents that may be degraded by bacteria already within the landfill, but it also contains ammonia at high concentrations (Kjeldsen et al. 2002), heavy metals, and other refractory organic and inorganic compounds that may accumulate in it, inducing bio-toxicity or bio-inhibition (Renou et al. 2008; Karrer et al. 1997).

Collected leachate is typically hauled to off-site treatment facilities, where it may interfere with biological processes due to heavy metal content, high ammonia concentration, or the presence of other xenobiotic pollutants (PAHs, organic halogens, PCBs) that may be refractory, inhibitory, or otherwise affect such processes (Callegari and Capodaglio 2017). Leachate may also present unbalanced C/N ratio content (especially in leachates from closed landfills), making it poorly biodegradable, and affect other processes due to its physical-chemical characteristics, e.g., reducing ultraviolet disinfection effectiveness by quenching UV light. All these factors may represent a major ordeal for many conventional treatment facilities, often requiring specific pretreatment. On-site pretreatment units could be specifically designed to address these needs, or even full treatment for subsequent discharge to municipal sewers; however, this may often not turn out as cost-effective. The most common processes for leachate treatment are biological (aerobic or anaerobic) and/or physicochemical, depending on pollutant content. “Emerging” technologies may also be appropriate (Wiszniowski et al. 2006). These include chemical oxidation (Kim and Huh 2009); adsorption (Foo and Hameed 2009); ammonia removal by biodegradation (Capodaglio et al. 2016b) or stripping (Cheung et al. 1997); evaporation, filtration, and reverse osmosis (Di Palma et al. 2002); sonication (Nazimudheen et al. 2018); Advanced Oxidation Processes (Capodaglio 2018, 2019) and others (Capodaglio 2017), depending on leachate composition, and discharge or site-specific constraints. Significant treatment efficiency improvement and decrease of overall treatment costs could be pursued by process combinations, to improve biodegradation of refractory organics (Koh et al. 2004; Geenens et al. 2001; Cecconet et al. 2017).

The sustainability of treatment processes in terms of energy input and related environmental emissions is becoming an issue of increasing relevance (Capodaglio and Olsson 2020); therefore, related considerations are becoming key discriminants in the choice of technology to be adopted, favoring those that can lead to reduction of either. Microbial fuel cells (MFCs) couple organic matter removal and energy recovery by direct conversion of the chemical energy in the substrate into electrical energy (Li et al. 2011; Capodaglio et al. 2013; Saba et al. 2017). MFCs have been pointed out as a promising bioelectrochemical technology for various types of liquid waste streams, including domestic (Ahn and Logan 2010) or industrial (Molognoni et al. 2018) wastewaters, and contaminated groundwater (Cecconet et al. 2020; Cecconet et al. 2018a). They were also indicated as an appropriate technology for landfill leachate treatment (Puig et al. 2011). The process is carried out by electrochemically active bacteria (EAB) that oxidize organic substrate in an anodic chamber, releasing electrons and protons (Logan et al. 2006). Electrons travel through an external electric circuit from the anode to the cathode, while protons pass directly through an ionic selective membrane to reach the cathode. There, both electrons and protons are recombined with the terminal electron acceptor (TEA), such as oxygen or nitrate (Logan and Rabaey 2013). MFC performance can be affected by several factors, such as substrate type and concentration, electrode material and surface area, ionic strength, pH, and cell design (Capodaglio et al. 2015; Cecconet et al. 2018b). Selected operating conditions may be exploited to optimize the structure of the cells’ microbiome (Molognoni et al. 2016) and improve bioelectrochemical efficiency (Capodaglio et al. 2017). MFCs have been used to treat easily biodegradable industrial wastewater (Callegari et al. 2018) and difficult-to-treat substrates (Abbasi et al. 2016; Srikanth et al. 2016). In the latter cases, like in any other biologically mediated processes, biomass acclimation to the specific pollutants is a key element for success (Capodaglio et al. 2010). The advantages of this type of technology are low energy inputs and the possibility of direct energy recovery, both strongly dependent on system architecture and operating conditions (Ge et al. 2014; Cecconet et al. 2018c).

Landfill leachate as a substrate for MFCs has been investigated under different circumstances (Hu et al. 2017; Huang et al. 2018; Li and Chen 2018; Zhang et al. 2015a; Zhang et al. 2015b) either alone or in combination with other processes (Mahmoud et al. 2014; Vázquez-Larios et al. 2014). Bioelectricity generation by MFCs creates additional opportunities for resource recovery from substrates, including leachate. While organic compounds are directly converted to electrical energy, nutrients (e.g., ammonia) can be recovered via migration and ammonium conversion at high pH resulting from the cathodic reduction (Iskander et al. 2016). Metals may also be removed or recovered by bioelectrochemical systems (Cecconet et al. 2018d). It was also shown that MFCs could produce an effluent water fit for irrigation reuse (Abourached et al. 2016). Addition of a readily biodegradable co-substrate is a common strategy to biologically treat substrates normally not suitable to biological processes, and increase overall process efficiency (Luo et al. 2009).

Simultaneous treatment of landfill leachate and wastewater with MFCs had been explored previously. Hernández-Flores et al. (2017) reported the combined treatment of leachate and municipal wastewater by adding 30, 50, and 70% of highly biodegradable leachate in the mixture, in this case presence of an increased biodegradable organic matter (leachate) enhanced electricity production. However, few studies dealt with leachates characterized by low biodegradability so far. In this study, mature leachate from a closed landfill, together with agro-industrial (dairy) wastewater as co-substrate, was fed to two differently structured dual-chamber MFCs at varying dilution ratios, to evaluate system performance and overcome process limitations connected to the poor biodegradability of a mature leachate as substrate for bioenergy production. The study also examined the MFC differential behavior in terms of electrodes’ performance, highlighting differences between the two tested materials for their construction. This study brings further insight in the treatment possibility of poorly biodegradable landfill leachate combined with highly degradable organic substrates with the use of bioelectrochemical systems.

## Materials and methods

### System setup and operation

**Fig. 1:**
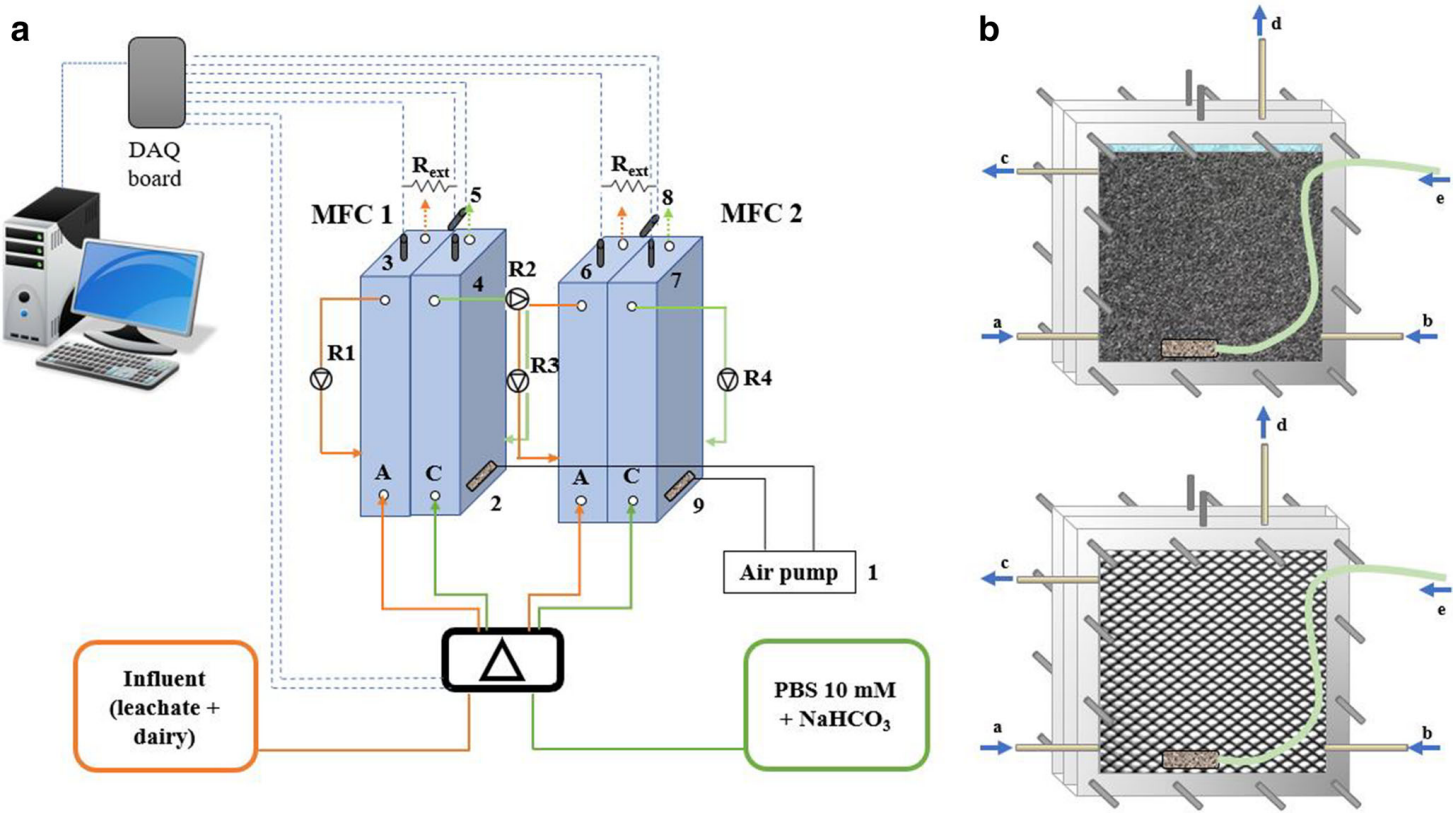
a Hydraulic and electrical connections. Continuous lines: hydraulic connections (anode: orange lines, cathode: green lines), R1, R2, R3, R4 recirculation pumps; dashed lines: electrical connections to DAQ board; dotted lines (green and orange): effluent discharge. (1) air pump; (2, 9) porous diffuser; (3, 6) anode electrode; (4, 7) cathode electrode; (5, 8) reference electrode. b Cathode chamber setup: MFC2 filled with granular graphite, MFC1 with stainless steel graphite-coated mesh. (a) inlet; (b) recirculation inlet; (c) recirculation outlet; (d) outlet; (e) air inlet

Two dual-chamber MFCs, each consisting of an anodic and a cathodic chamber on the opposite sides of a methacrylate rectangular frame, separated by a cationic exchange membrane (CEM, CMI-7000, Membranes International Inc., USA), were operated and closely monitored during the study. The two structurally identical cells (from now on, indicated as MFC1 and MFC2) differed only for the constituting electrode material. MFC1 was built with graphite-coated stainless steel (GCSS) mesh (200 × 200 mm sheets) electrodes in both chambers, while MFC2 anodic and cathodic chambers’ electrodes were made of granular graphite (model 00514, diameter 1.5–5 mm, EnViro-Cell, Germany). The final free volume of each chamber was 800 mL (net anodic chamber, NAC, and net cathodic chamber, NCC) in MFC1 and 450 mL (NAC and NCC) in MFC2, respectively. In order to allow external circuit connection, graphite rod electrodes (250 × 4 mm) were inserted in both chambers. A 33 Ω resistance was connected to MFC’s external circuit: this value was determined to be as close as possible to the static internal resistance of the MFCs. An Ag/AgCl reference electrode was placed in the anodic chamber (+ 197 mV vs SHE, Xi’an Yima Opto-Electrical Technology Co., China). Oxygen was the terminal electron acceptor, provided directly into the cathodic chambers by a porous diffuser connected to a fish tank air pump. The scheme of the experimental system is shown in Fig. 1.

Influent dosage and recirculation were controlled by peristaltic pumps (BT100N, Baoding Shenzhen Precision Pump Co., China) connected to a pre-programmed controller. Close-circuit recirculation was operated continuously to accomplish well-mixed conditions within anodic chambers; influent flow rate was set at 1 L day^−1^ in step-feeding mode (20 min each hour). The two MFCs were inoculated with a mixture of activated sludge and effluent of a parent MFC treating only dairy wastewater (DW).

A mixture of DW and screened leachate from a nearby landfill was fed to the anodic chambers during the study. Landfill leachate (LL) and DW characteristics are reported in Table 1. The formers were constant throughout the study (resulting from a one-time sample collection), while DW was collected weekly, due to its quick biodegradability, with quality varying slightly during the study, due to the different process cycles operated at the cheese factory. Both landfill leachate and dairy wastewater were stored at 4 °C after collection and until use. Phosphate buffer solution (PBS, 10 mM, pH = 7) was used as pH-control medium for the cathodic chamber, with the following composition: 507 mg L^−1^ NaH_2_PO_4_, 819 mg L^−1^ Na_2_HPO_4_, 1000 mg L^−1^ NaHCO_3_, 130 mg L^−1^ KCl, 310 mg L^−1^ NH_4_Cl (Xia et al. 2013).

**Table 1:** Main characteristics of leachate and dairy influent

Parameter	Units	Leachate	Dairy (range during study)
pH		8.28	5.5–8.9
Electric conductivity (20 °C)	mS/cm	22.1	9–16
COD	mg/kg	2420	1150–2670
BOD_5_	mg/kg	215	710–1230
NH_4_^+^	mg/kg	2595	8–23
N-NO_2_^-^	mg/kg	< 1	2–9
N-NO_3_^-^	mg/kg	16.8	7–21
P_tot_	mg/kg	158	30–84
Total suspended solids	mg/kg	41	42–170
Heavy metals	mg/kg	Traces	Not tested

### Data collection and evaluation

Anodic potentials were monitored with an Ag/AgCl reference electrode and continuously acquired at 1-min intervals by an automated data acquisition system (NI USB-6008, National Instruments Italy) connected to a computer. MFCs’ generated voltages (*V*) were simultaneously recorded. Power (*P*) and current (*I*) were determined from continuous voltage measurement. Current (dI) and power (dP) densities were calculated dividing the respective value of *I* and *P* by the NAC volume of each cell. Anodic coulombic efficiency (CE) was computed using daily average data of flow rate and current intensity.

Determination of effluent COD (daily composite samples for each cell) and influent wastewater COD (one sample for every batch collected) was performed according to the “standard methods” (APHA 2017). Anodic organic loading rate (OLR) was calculated as the daily organic matter concentration (in terms of COD) divided by the anode’s hydraulic retention time (HRT). Organic matter removal efficiency (ηCOD, percent) was determined as described in Molognoni et al. (2014). Conductivity and pH were measured at least once every 5 days for both anode and cathode influents and effluents (IntelliCAL^TM^ probes + HQd^TM^ Digital Meter, Hach Lange).

The normalized energy recovery (NER) of the MFCs, a parameter that expresses the amount of energy recovered per removed mass of COD (NER_S_, kWh kg COD_removed_^−1^) and per volume of treated wastewater (NER_V_, kWh m^−3^_treated_), was calculated for each period and for the total experiment with the following equations, as proposed by Ge et al. (2014):

1 $$ {\mathrm{NER}}_{\mathrm{V}}=\frac{P\cdotp t}{{\mathrm{V}}_{\mathrm{treated}}} $$

2 $$ {\mathrm{NER}}_{\mathrm{S}}=\frac{P\cdotp t}{{\mathrm{kg}}_{\mathrm{CODremoved}}} $$

Construction of polarization (V, I) and power curves (V, P).was also performed by using a potentiostat (NEV 4, Nanoelectra, Spain) to verify the internal resistance of the system and identify differences between the two setups and analyze energy losses.

### Experimental procedure

The experimental study was divided into 11 successive phases, each operated for 1 week, a period necessary for achievement of a stable electrical production. Cell inoculation occurred running the systems using the effluent of a parent MFC and sewage sludge of dairy origin as influent substrates, until establishment of a suitable microbiome was observed. In phase 0, both MFCs were fed with dairy wastewater only, afterwards—during phases 1–10—the feed consisted of a mix of LL and DW at increasing ratios, with 5% step increase of LL at each subsequent phase. The main characteristics of the influent feed during the study are reported in Table 2.

**Table 2:** Characteristics of anodic influent throughout the study

Study phase	Leachate (%)	OLR (kg COD m^-3^ day^−1^)	pH,_IN_	Conductivity,_IN_ (mS/cm)
Phase 0	0	1.49	7.85	1.99
Phase 1	5	1.16	7.42	4.16
Phase 2	10	0.87	8.72	2.74
Phase 3	15	2.39	7.15	4.64
Phase 4	20	2.14	8.17	5.23
Phase 5	25	1.15	7.94	4.64
Phase 6	30	0.71	7.99	5.02
Phase 7	35	1.20	8.38	5.03
Phase 8	40	1.19	7.33	5.61
Phase 9	45	1.34	8.24	6.20
Phase 10	50	1.34	8.00	6.75

## Results and discussion

### Electric production

Microbial fuel cells rely on biological oxidation of wastewater, which effectiveness strongly depends on the nature of the substrate. LL used in the present experimentation is a poorly biodegradable substrate; to enhance its suitability for biological treatment DW, a highly biodegradable substrate was used as co-substrate.

**Fig. 2:**
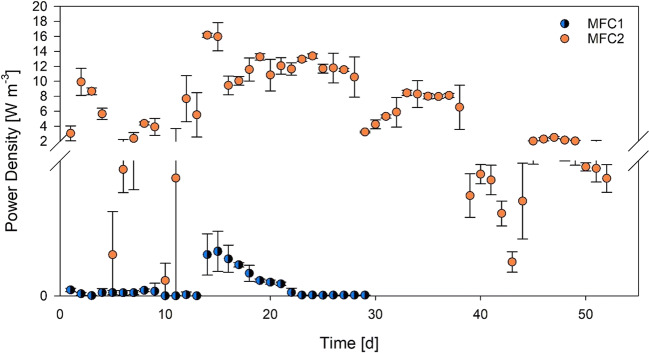
Power density monitored throughout the experimentation. Error bars report the power range monitored each day

Observed energy production did not reflect a specific trend correlated to the varying LL fraction in the feed; however, upon examination of the results, it can be assessed that the most favorable operating condition was observed in phase 4 (15% leachate), where maximum output power peaks were recorded for both MFCs. It must be stressed out that the characteristics of leachate remained constant during the study, while DW parameters changed slightly, as previously shown in Table 1, although previous studies on substrates from the same source showed consistent excellent degradability and energy production when fed to similar MFCs (Callegari et al. 2018). Maximum voltage achieved for MFC1 and MFC2 was 151.1 mV and 509.3 mV, respectively, corresponding to current densities of 4.6 and 15.4 A m^−3^. Power density monitored throughout the experimentation is represented in Fig. 2. MFC1 showed much lower electrical production than MFC2 throughout the whole study, highlighting how important factors such as setup design and adopted materials affect this systems’ performance. MFC1 maintained fair power generation throughout phases 3 and 4, dropping considerably during phase 5 (voltage measured between electrodes stabilized at around 10 mV). MFC2 maintained, instead, higher and stable values of electrical production up to phase 7, after which measured voltage dropped to below 170 mV (corresponding to current density of 5 A m^−3^) under all subsequent operating condition tested.

In both systems, after the shift from DW-only feed to the 5% LL-DW mix, an instantaneous drop in energy production was observed, which could be attributed to ongoing acclimation of the MFCs’ anodic biomass to the new substrate composition. This acclimation is confirmed by the rapid recovery observed in the following days, with rapid exoelectrogenic biomass activity recovery, which maintained and improved high current production throughout phases 2 and 3 for MFC1, and up to phase 7 for MFC2, even at increasing leachate ratios in the feed.

At this point, it seems evident that MFC2 architecture proved to be more efficient for energy recovery than MFC1’s as, both being operated under the same conditions, the latter showed a consistently lower power generation.

### Electric and organic matter removal efficiency

**Fig. 3:**
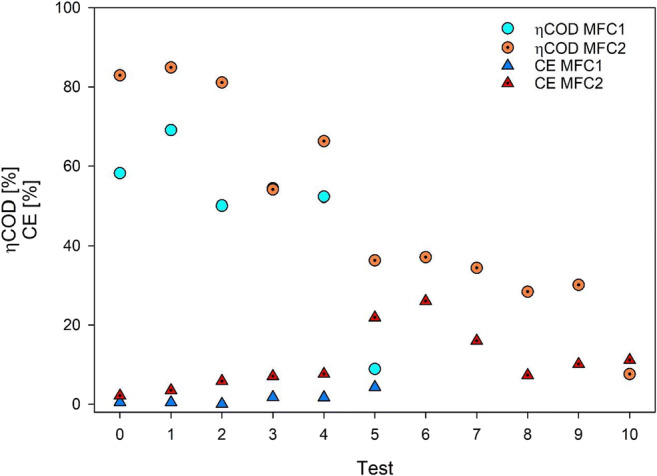
CE and ηCOD in MFC1 and MFC2

ηCOD throughout the study was measured for each condition tested, and CE was calculated. In the first phases of the study, CE was very low for both systems, probably due to slow adjustment of the exoelectrogenic population to the substrate. Concerning MFC1, CE showed a linear incremental trend (Fig. 3), with values ranging from 1 to 6% in the last condition tested, while MFC2 showed more variability, with sudden increase under phases 5 and 6, where the maximum efficiency (26%) was observed, decreased down to around 10% afterwards.

COD removal efficiency started at 82.9% for MFC2 and 58.1% for MFC1 in phase 0. It increased in phase 1, achieving the best values for both MFCs, 84.9% and 69.1% for MFC2 and MFC1, respectively, decreasing gradually with the increase of leachate ratio in the feed. During phase 5, COD removal dropped drastically in MFC1, at 7.6%. The unit was then operated until the end of phase 6, with no increase in voltage generation and even lower ηCOD, at 5.7%; therefore, it was decided to stop the operation of this unit. MFC2 maintained high COD removal efficiency (generally at or above 66%, save for a low of about 55% during phase 3) until phase 5. At 25%, LL ratio in the feed conditions became critical: from the previous ηCOD of 66.3%, removal dropped by almost half to 36.5%. This content level of landfill leachate in the influent affected both systems and thus can be considered their operational limit in the studied conditions. MFC2 maintained, however, removal efficiency greater than 30% until phase 10 (LL/DW = 50%), when ηCOD dropped to a low of 8.6%.

### Polarization curves

**Fig. 4:**
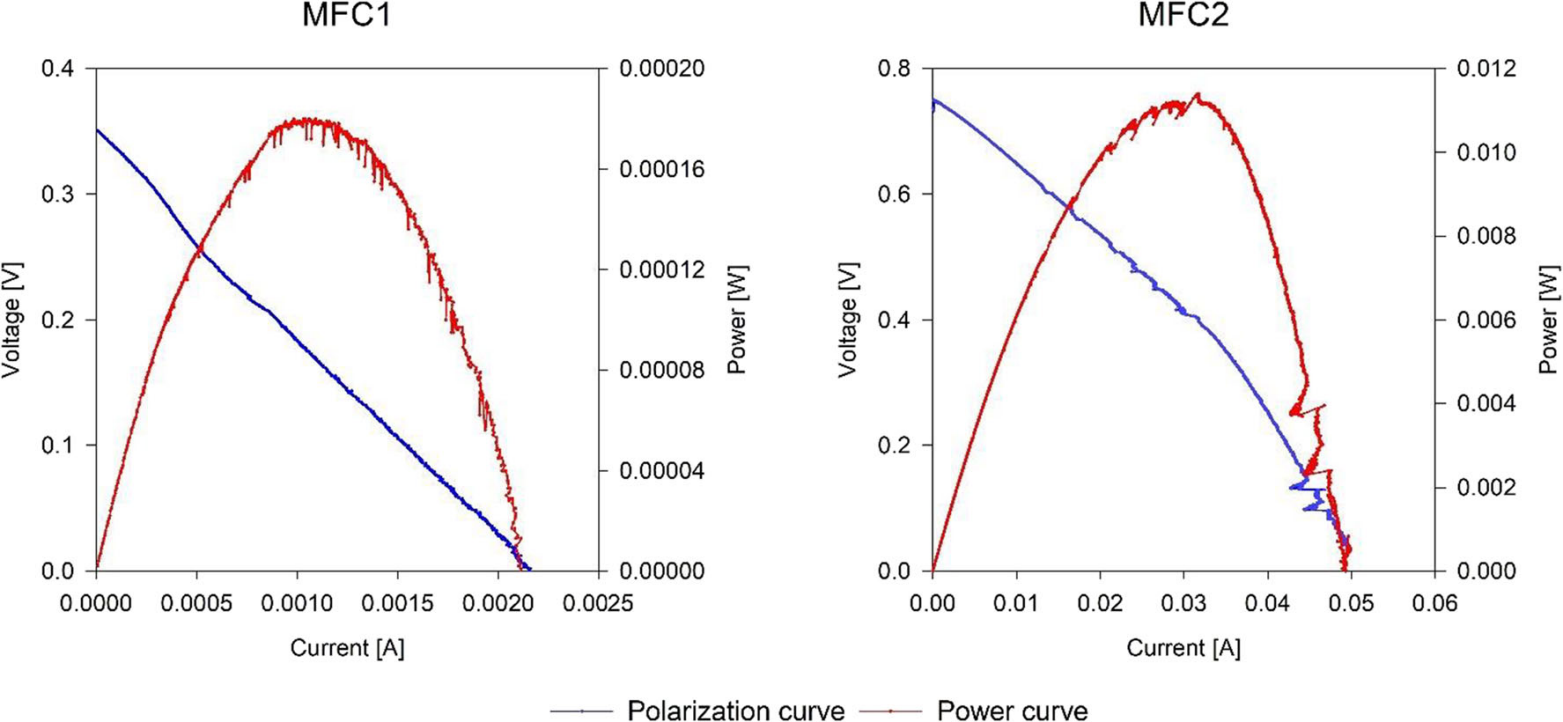
Polarization and power curves performed during phase 2

A final analysis concerned the systems’ polarization and power curves: in addition to representing the electrical behavior of the cells, they allow to establish the real internal resistance value; it was already reported that, to maximize energy production in MFCs, external resistance should be equal to the internal one (Molognoni et al. 2016). Polarization and power curves (Fig. 4) were determined for each experimental condition: early examination of the observed power curves of the MFCs showed that MFC2’s internal resistance was 21 ± 10 Ω, quite close to the external resistance actually applied (33 Ω), while MFC1’s internal resistance resulted in a staggering 170 ± 18 Ω, five times higher. This difference is largely due to the electrodes constituting materials of the cells and justifies both the initial lower power generation and CE of the first unit. After phase 2, the external resistance of MFC1 was modified to 150 Ω, showing a detectable increase in power density, although no direct benefit was seen in COD removal efficiency during subsequent tests. This modification did not prevent the system to substantially stop being efficient in terms of COD removal and energy recovery between phases 5 and 6.

The internal resistance detected for MFC2, instead, was similar to the external resistance initially applied; therefore, further analysis of energy losses in the unit was performed. It was found that the largest part (*E*_t_ = 55%) of these could be attributed to membrane losses, while the second largest factor affecting energy production was cathode efficiency (*η*_cat_ = 32%). Anode efficiency and pH gradient only accounted for 7% and 5% loss respectively, while ionic exchange between anode and cathode could be considered negligible (< 1% loss).

### Comparative analysis

**Fig. 5:**
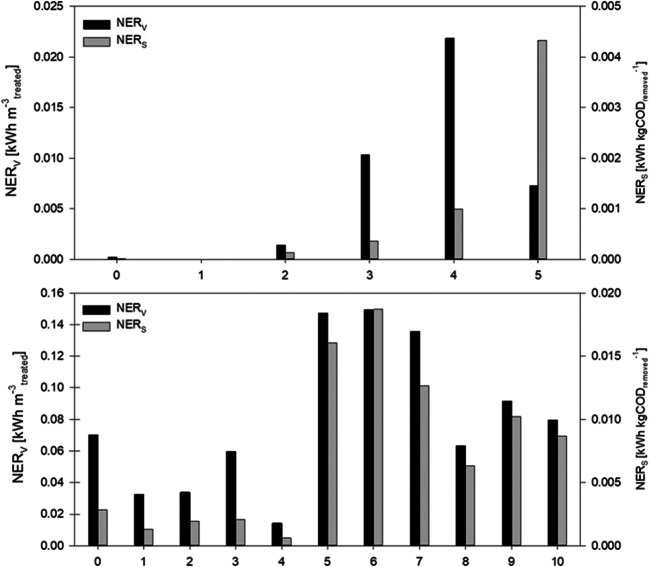
NER_V_ and NER_S_ obtained throughout the study: MFC1 (upper) and MFC2 (lower)

NER throughout the study was evaluated for both units, in volumetric (NER_v_, net energy recovery per m^3^ influent treated) and massive (NER_S_, net energy recovery per kg COD removed) specific terms. Results are summarized in Fig. 5: it can be noticed that it was not possible to establish a consistent trend of this parameter in relationship with observed COD removal and CE. MFC1 (Fig. 5, upper) recovered almost no energy during the first tests, due to suboptimal electric circuit conditions. When sufficient energy production started (phases 3 and 4), values up to 0.022 kWh m^−3^_treated_ were observed. As already confirmed by the previously shown data, MFC2 showed better performance, reaching values of NER_V_ of 0.149 kWh m^−3^_treated_ during phase 6 (30% leachate). In terms of specific net energy recovery, the best rates were also obtained in phase 6, with NER_S_ of 0.019 kWh kg COD^−1^.

To compare the results of the present study to others reported in literature, phase 4 was taken as reference for both units tested. Reported studies taken for comparison are summarized in Table 3. When considering landfill leachate as a substrate, the type of landfill, age, and wastes collected strongly influence performance of a bioelectrochemical system and must be taken into account. Also, pretreatment increase the bioavailability of organic matter in leachate, for example, by performing fermentation, enhancing electricity production and substrate conversion (Mahmoud et al. 2014). Along with COD removal, in many studies, nutrients’ removal, such as ammonia and phosphorus, was evaluated. However, not being the main focus in the present work, these were not taken into account for the comparison. Fresh landfill leachate normally has relatively high BOD_5_/COD ratio (0.4–0.6) indicating good biodegradability (Özkaya et al. 2014). This ratio generally decreases with the age of the landfill: the present study operated on leachate from a closed landfill, characterized by a low BOD_5_/COD ratio of about 0.1.

**Table 3:** Net energy recovery from landfill leachate bioelectrochemical systems applications (NER_V_ and NER_S_ calculated according to Iskander et al. (2016))

System configuration	Leachate COD (mg L^−1^)	Operational mode	COD removal (%)	CE (%)	NER_V_ (kWh m^−3^_tr_)	NER_S_ (kWh kg_CODrem_^−1^)	Reference
Membrane-less anoxic/oxic	19,200	Continuous	95.1	-	-	0.04866	Zhang et al. (2015a)
Dual chamber	50,000	Continuous	43	< 1.0	0.05400	0.00251	Özkaya et al. (2014)
Single chamber	12,300	Batch	72	6.7	-	0.01986	Vázquez-Larios et al. (2014)
Single chamber (air cathode)	507 (diluted)	Continuous	32	< 2.0	0.0000506	0.00031	Puig et al. (2011)
Membrane-less anoxic/oxic	20,100	Continuous	86	-	0.06648	0.00383	Zhang et al. (2015b)
Dual chamber	11,400	Continuous	87	0.6	-	0.00190	Zhang and He (2013)
Dual chamber	300 (diluted 15%)	Continuous	26	-	-	-	Nguyen and Min (2020)
Dual chamber	4000 (synthetic)	Batch	65.1	-	-	-	Huang et al. (2018)
Dual chamber	2216	Step-feed	53.6	6.9	0.0068	0.0058	Present study (MFC1)
Dual chamber	2216	Step-feed	56.2	13.5	0.074	0.00714	Present study (MFC2)

Puig et al. (2011) operated an air cathode MFC with both diluted and raw landfill leachate characterized by low BOD_5_/COD ratios (0.02–0.2) and high salinity, comparable with that used in the present study. During operation with diluted leachate (507 mg COD L^−1^, OLR = 1.48 kg COD m^−3^), an air cathode MFC achieved 32% COD removal, and average power density of 6.1 ± 4.2 mW m^−3^. With raw leachate fed to the system, OLR increased up to 24.42 kg COD m^−3^, achieving up to 37% COD removal and power density of 344 mW m^−3^. Observed coulombic efficiency, however, remained below 2%, indicating that substrate degradation was not carried out primarily by exoelectrogenic bacteria, but possibly by methanogens, a commonly found EAB-competing species (Molognoni et al. 2016).

Most MFC studies in literature concern the use of fresh landfill leachate: this is, in fact, easily biodegradable, leading to an easier and more effective biological treatment, but not necessarily to higher energy recovery efficiency. Özkaya et al. (2014) operated an MFC with such substrate, characterized by COD up to 50 g L^−1^ (BOD_5_/COD = 0.65), starting from COD concentration of 1 g L^−1^, and reducing gradually the applied OLR up to 50 g L^−1^ day^−1^. Higher OLRs led to lower coulombic efficiency (< 1%, against 35% at lower ORLs). The authors stated that, despite the overall increase in voltage output, decrease in CE may be due to uptake of organics by non-exoelectrogenic processes, such as methanogenesis. Zhang et al. (2015a, b) operated dual-chamber BESs for fresh landfill leachate treatment, characterized by BOD_5_/COD = 0.48, achieving 2.16 W m^-3^ maximum energy recovery and 95.1% COD removal at OLR of 1.2 kg COD m^−3^ day^−1^. These are the best performance values reported so far in literature. Vázquez-Larios et al. (2014) operated MFCs with fresh landfill leachate with excellent biodegradability (BOD_5_/COD = 0.86) in a two chambered MFC in batch mode. COD removal of 72% was achieved, with maximum power density of 1.83 W m^−3^.

The present study shows that both units (MFC1 for part of the tests only), even though fed with diluted old, low biodegradability landfill leachate, achieved satisfactory degradation values and energy recovery parameters in line with those reported in literature for any type of leachate. It should be also noted that not all published studies examined clearly specify the period during which the observed performances were consistently maintained.

### End of operation analysis

**Fig. 6:**
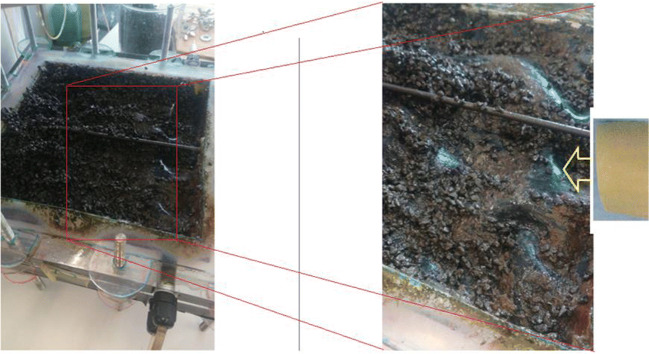
Solid residues observed between the electrode’s graphite granules in the open anodic chamber of MFC2 after the study. The black sheet material indicated by the arrow in the right panel is the cell’s CEM (shown in new original condition in the picture rightmost insert)

To better understand the limitations of landfill leachate treatment, and the causes that led to failure of the process when the ratio LL/DW = 1 (50%) in the feed was reached, an autoptic analysis was performed on the cells at the end of the study. After conclusion of the tests, both MFCs were disassembled to analyze the effects of the continuous operation with landfill leachate mix feed on the constituent materials. Figure 6 shows actual photographs of the anodic chamber of MFC2, indicating solid particles obstructing the spaces between the electrode’s graphite granules, limiting contact possibility between substrate and electrode surface. Notwithstanding a preliminary screening of the leachate performed upon collection, the constant flow of raw landfill leachate, in which colloidal and small solid, non-biodegradable particles may have remained, caused their gradual accumulation in the anodic chamber, reducing its net free volume in time, and consequently its hydraulic retention time, affecting the systems’ overall performance. The effect of internal hydrodynamic conditions and flow distribution on cell performance had already been highlighted in literature (Cecconet et al. 2018b; Vilà-Rovira et al. 2015), and this additional evidence confirms previous findings. In addition, non-pretreated landfill leachate could also have caused partial fouling of the CEM, affecting ion transfer efficiency between chambers, and decreasing overall performance of the unit (Xu et al. 2012). Finally, the presence of trace metals and ammonia may also have affected MFC performance with a potential biomass inhibiting effect (Hang et al. 2020).

## Discussion

Results of the study showed that one of the MFCs tested for combined leachate and industrial wastewater treatment obtained initially good results both in terms of COD removal and power generation. The use of DW as co-substrate provided additional nutrients to the EABs and resulted in improved bioelectrochemical degradation of organics, compared with feed with LL only. The unit that achieved the best performance (MFC2) had electrodes built with granular graphite, while the one (MFC1) with GCSS mesh electrodes showed poor performance since start-up. As pointed out by several studies, the performance of MFCs in terms of power output and durability strongly depends on the key components of these systems, the electrodes, which are one of the limiting factors for a generalized applicability of these systems (Gnanakumar et al. 2013). Anode and cathode material research is among the most active sector in bioelectrochemical systems, together with unit scalability issues (Abdallah et al. 2019). Premature failure of MFC1 could be ascribed to the poor performance of the GCSS mesh electrode material in these conditions. The performance of the granular graphite unit was satisfactory, comparable with that of most similar literature reported studies, until process deterioration, mostly due to physical clogging within the anodic compartment, occurred.

Some of the clogging problems detected during this study could be solved by adequate pretreatment of landfill leachate: more particle-selective influent screening should be implemented, possibly in combination with improved cell electrode design allowing efficient free circulation of residual particulate material within the cell. Pretreatment could also be considered in order to enhance leachate biodegradability. Ultrasonication, for example, was shown to increase soluble COD fractions and modify leachate composition in terms of NH_3_-N and acetate concentrations (Nazimudheen et al. 2018). High ammonia levels may stripped by air and calcium hydroxide, removing up to 70% of leachate’s ammonia content (Cheung et al. 1997). Fermentation processes prior to bioelectrochemical treatment was also reported to enhance MFC power recovery, with organic removal improvement by up to 15 times (Mahmoud et al. 2014).

## Conclusions

Two MFCs were operated for treatment of combined poorly biodegradable (BOD/COD = 0.1) landfill leachate and dairy wastewater as co-substrates at various mixing ratios. Both units, with similar architecture but different electrode constituting materials and net cell volumes, were operated under continuous feed. MFC1 was operated for 6 cycle phases, up to 25% leachate percentage in the feed, while MFC2 maintained residual efficiency until reaching a feed composition of 50% leachate, prior to process failure. Both systems achieved their best performance treating a mixture of 20% leachate and 80% dairy wastewater. Premature failure was ascribed to poor electrically performing anodic material in the first cell. As far as the second cell, after a posteriori autoptic examination of the unit, failure was ascribed to accumulated interference of feed-contained solids, which determined clogging of the anode cell free volume in time, favored by suboptimal internal hydrodynamic conditions. Pretreatment of leachate may be the key to operate at higher percentages in the influent solution, lowering the presence of residual non-biodegradable solids or inhibiting waste components. Despite the ultimate process failure, during the first stages of the study, MFC2 performance was quite similar to that reported by other studies.

Bioelectrochemical systems have shown consistent sustained, long-term treatment performance of different substrates and good short-term treatment performance of problem substrates such as landfill leachate, especially when fed with fresh leachate. Further attempts in this direction should consider adequate substrate pretreatment or internal hydrodynamic improvements to overcome the drawbacks observed in this study, in particular when aged, poorly biodegradable leachate is fed as substrate.
